# Functional studies of novel *CYP21A2* mutations detected in Norwegian patients with congenital adrenal hyperplasia

**DOI:** 10.1530/EC-14-0032

**Published:** 2014-04-15

**Authors:** Ingeborg Brønstad, Lars Breivik, Paal Methlie, Anette S B Wolff, Eirik Bratland, Ingrid Nermoen, Kristian Løvås, Eystein S Husebye

**Affiliations:** 1 Department of Clinical Science University of Bergen 5021, Bergen Norway; 2 Department of Medicine Haukeland University Hospital Bergen Norway; 3 Division of Medicine Akershus University Hospital Lørenskog Norway

**Keywords:** *CYP21A2*, congenital adrenal hyperplasia, functional studies, novel mutations

## Abstract

In about 95% of cases, congenital adrenal hyperplasia (CAH) is caused by mutations in *CYP21A2* gene encoding steroid 21-hydroxylase (21OH). Recently, we have reported four novel *CYP21A2* variants in the Norwegian population of patients with CAH, of which p.L388R and p.E140K were associated with salt wasting (SW), p.P45L with simple virilising (SV) and p.V211M+p.V281L with SV to non-classical (NC) phenotypes. We aimed to characterise the novel variants functionally utilising a newly designed *in vitro* assay of 21OH enzyme activity and structural simulations and compare the results with clinical phenotypes. *CYP21A2* mutations and variants were expressed *in vitro*. Enzyme activity was assayed by assessing the conversion of 17-hydroxyprogesterone to 11-deoxycortisol by liquid chromatography tandem mass spectroscopy. PyMOL 1.3 was used for structural simulations, and PolyPhen2 and PROVEAN for predicting the severity of the mutants. The *CYP21A2* mutants, p.L388R and p.E140K, exhibited 1.1 and 11.3% of wt 21OH enzyme activity, respectively, *in vitro*. We could not detect any functional deficiency of the p.P45L variant *in vitro*; although prediction tools suggest p.P45L to be pathogenic. p.V211M displayed enzyme activity equivalent to the wt *in vitro*, which was supported by *in silico* analyses. We found good correlations between phenotype and the *in vitro* enzyme activities of the SW mutants, but not for the SV p.P45L variant. p.V211M might have a synergistic effect together with p.V281L, explaining a phenotype between SV and NC CAH.

## Introduction

The *CYP21A2* gene encodes the enzyme steroid 21-hydroxylase (21OH), which is essential for steroid synthesis in the adrenal cortex. Mutations in *CYP21A2* are the main cause of the autosomal recessive disorder congenital adrenal hyperplasia (CAH) (1). The classical forms of CAH are the salt wasting (SW) and simple virilising (SV) phenotypes with adrenocorticotropic hormone-driven excess of androgens leading to ambiguous genitalia in female newborns. The SW phenotype has complete or almost complete loss of enzyme activity, leading to lack of cortisol and aldosterone, whereas the SV displays various degrees of reduced enzyme activity resulting in cortisol insufficiency (1, 2). The non-classical (NC) CAH is milder and characterised by signs of hyperandrogenism postnatally and in adulthood, and associated with minor mutations (3).

The most common mutations in the *CYP21A2* gene are derived from a non-functional pseudogene *CYP21A1P* (4). Both *CYP21A2* and *CYP21A1P* are located in the RCCX module of the HLA class III locus of chromosome 6 and share about 98% sequence homology. In general, there is a high correlation between the *CYP21A2* genotype and the CAH phenotype (5, 6). In addition to the pseudogene mutations, more than 160 other pathological variants of *CYP21A2* have been reported (www.cypalleles.ki.se/cyp21.htm).

In the present study, we used a new *in vitro* assay of 21OH enzyme activity and molecular computer modelling to predict the phenotypes of recently detected variants (p.L388R p.E140K, p.P45L and p.V211M) in the Norwegian population (7). We also checked a novel variant (p.A159T), detected in Norwegian patients with autoimmune Addison's disease (AAD) and in control populations.

## Subjects and methods

### Subjects

We detected four novel variants: p.L388R, p.E140K, p.P45L and p.V211M, in a cohort of 59 patients diagnosed with CAH (7). Four unrelated female patients, each had one of the missense variants listed above, except the subject with p.V211M who also had a coupled p.V281L mutation on the same allele. All patients exhibited a deletion in the other allele (7). Clinical characteristics of the patients are listed in Table 1. The fifth variant, p.A159T, was found in heterozygosity in three of 370 patients with AAD and in one out of 330 Norwegian healthy subjects.

### Genetic analyses of *CYP21A2*

Protocols for sequencing and copy number analyses of the *CYP21A2* gene were described previously (7, 8, 9, 10, 11).

### Mutagenesis

Stratagene's QuikChange II Site Directed Mutagenesis Kit (Agilent Technologies, Santa Clara, CA, USA) was used to introduce point mutations in the pcDNA2 plasmid containing *CYP21A2* cDNA (a kind gift from Prof. Holger Luthman, Department of Molecular Medicine, Karolinska Institute, Stockholm, Sweden (12)), corresponding to the novel *CYP21A2* variants (p.L388R, p.E140K, p.P45L, p.V211M and p.A159T). Three control variants of *CYP21A2* were also generated: p.R102K (normal variant), p.I172N (SV phenotype) and p.G291R (SW phenotype). Primers used for mutagenesis are listed in Table 2. The mutagenesis was carried out according to the manufactures protocol, using 16 cycles with 5 min extension time. The mutagenesis was verified with direct sequencing, and plasmids from positive clones were purified by HiSpeed Plasmid Maxi Kit (Qiagen).

### *In vitro* protein expression of 21OH mutants

Non-radioactive TNT Quick Coupled Transcription/Translation system (Promega) with Sp6 RNA polymerase was used for *in vitro* protein expression. Expression of all the 21OH variants was verified by western blot using a primary antibody against 21OH (c17, goat polyclonal IgG, Santa Cruz Biotechnology) and alkaline phosphatase-conjugated donkey anti-goat IgG secondary antibody (Santa Cruz Biotechnology).

### Relative quantitation of 21OH from *in vitro* expression

ELISA plates (MaxiSorp, Nunc Thermo Scientific, Waltham, MA, USA) were coated over night with 10 μl of the *in vitro* expression product diluted in PBS, pH 7.4. Primary antibody against 21OH (Santa Cruz Biotechnology), together with the corresponding alkaline phosphatase-conjugated secondary antibody (Sigma) and the FAST pNPP detection system (Sigma), was used to determine the relative amount of 21OH in the reaction products.

The ELISA method was verified by selected reaction monitoring (SRM) against two signature peptides of 21OH (p.85–98, LQEELDHELGPGASSSR and p.317–333, WADFAGRPEPLTYK). Briefly, 5 μl of each version of 21OH reaction products were taken out for quantitative determination of 21OH. The lysates were purified on SDS–PAGE gel, and the bands corresponding to the molecular weight of wt 21OH (56 kDa) were cut out for trypsin treatment. Stable isotope-labelled synthetic peptides (N-terminal replacement of lysine (^13^C_6_, ^15^N_2_) or arginine (^13^C_6_, ^15^N_4_) respectively) were added in constant amount before purification, resulting in a mass difference of 8 or 10 Da to the corresponding endogenous peptide. A Q-Trap 5500 (AB SCIEX) connected to a Dionex Ultimate NCR-3500RS LC system was used for the SRM analyses. The SRM data were analysed using Skyline, version 2.1 (https://skyline.gs.washington.edu/), and the most abundant transition was used for quantification. The SRM analyses were carried out using standard procedures by the Proteomics Unit (PROBE) at the University of Bergen. For quantitation, the amounts of 21OH constructs were calculated relative to the amounts of the 21OH wt protein.

### Enzyme activity assay

The activity of the different 21OH constructs was estimated by quantitating 11-deoxycortisol (11DOC) using 17-hydroxyprogesterone (17OHP) as substrate. The enzyme reactions were set up mainly as described previously (13). Briefly, from each 21OH construct, 50 μl of the *in vitro* expression product were mixed with 15 pmol of recombinant human cytochrome P450 oxidoreductase (CYPOR, Sigma–Aldrich) and 25 μl of 300 μM 17OHP (in 10% methanol) on ice. Pre-warmed reaction buffer (50 mM potassium phosphate (pH 7.4), 20% (v/v) glycerol, 2 mM dithiothreitol, 0.1 mM EDTA and 0.0034% (v/v) Emulgen 913 (Karlan Research Products Corporation, Cottonwood, AZ, USA)) was then added to a total volume of 500 μl. After 60 s of pre-incubation at 37 °C, the enzymatic reaction was initiated by adding 5 μl of 100 mM NAPDH. The enzymatic reaction was carried out for 1 h at 37 °C, and then terminated by putting onto ice. Further, the enzyme reaction solution was diluted 1:10 or 1:100 in a solution of 50% methanol and 0.1% formic acid in double-distilled (dd) H_2_O (v/v). An internal standard (IS) solution (50 nM 17OHP-d8 and 50 nM 11DOC-d2 in methanol) was then added to the diluted samples in a 1:1 ratio. Working standards of 11DOC in the range of 0.61–150 nM were prepared by diluting stock solutions in a solution of 50% methanol and 0.1% formic acid in ddH_2_O (v/v) and adding IS (1:1). Working standards and 11DOC produced from each enzymatic reaction were quantified by liquid chromatography coupled to tandem mass spectroscopy (LC–MSMS) (14). The enzyme activity was calculated as the % ratio of 11DOC production between the 21OH mutants to the wt, adjusted for the quantity of 21OH wt and mutant in the enzymatic reactions.

### Structural simulations and *in silico* analyses

The molecular graphic images were generated from a humanised version of bovine 21OH as template (15), kindly provided from Dr Shozeb Haider (Centre of Cancer Research and Cell Biology, Queens's University of Belfast, Belfast, UK), using The PyMOL Molecular Graphics System, version 1.3 (Schrödinger, LLC, Portland, OR, USA). Variants were modelled using the Mutagenesis tool in PyMOL and distances between molecules measured using the Measurement tool. The severity of the variants was predicted by PolyPhen-2 v2.2.2r398 HumVar model (http://genetics.bwh.harvard.edu/pph2/) (16) and PROVEAN (http://provean.jcvi.org/index.php) (17) using default settings. Sequence alignments of 21OH from seven different species (GenBank: AAB59440.1 (*Homo sapiens*), AAA30487.1 (*Bos taurus*), ABC69211.1 (*Felis catus*), AAD05573.1 (*Rattus norvegicus*) and BAF63009.1 (*Gallus gallus*); NCBI reference sequence: XP_003311237.1 (*Pan troglodytes*) and NP_999598.1 (*Sus scrofa*)) were carried out by default settings using Clustal omega (http://www.ebi.ac.uk/Tools/msa/clustalo/) (18) in order to assess the evolutionary conservation of the mutated amino acids.

### Statistical analyses

Statistical analyses were performed by the GraphPad Prism, version 5.02 Software (La Jolla, CA, USA). Correlations between the ELISA and SRM methods for quantifying the 21OH proteins produced were calculated by Pearson's correlation test. Deviation of enzyme activity of the 21OH mutants compared with the wt 21OH was calculated by ANOVA combined with Dunnett's multiple comparison tests using a significance level of *P*<0.05.

## Results

### Verification and relative quantitation of 21OH by ELISA and SRM methods

Western blot analysis of the samples from *in vitro* expression showed a 56-kDa band corresponding to the molecular weight of 21OH for all constructs (data not shown).

The ELISA method for quantitation of the mutated 21OH proteins correlated very well with the SRM method (*P*<0.0001, Pearson's *r*=0.9206, and 95% CI (0.8016, 0.9695), *n*=19). Thus, the ELISA method was used for further quantitation of 21OH.

### *In vitro* 21OH activity studies

The assay showed no significant reduction in the enzyme activity of the p.R102K normal variant relative to the wt (119.7%), whereas the p.I172N (SV phenotype) and p.G291R (SW phenotype) mutations showed significant (*P*<0.05) decline (1.6 and 0%, respectively, relative to wt (Table 3)). The novel CAH mutations, p.L388R and p.E140K, displayed significantly (*P*<0.05) reduced activity of 1.1 and 11.3% compared with wt respectively (Table 3). The other two novel CAH variants, p.P45L and p.V211M, were similar to the wt (105 and 99.5% respectively) as was p.A159T (126.6%) (Table 3).

### Structural simulations

**Figure 1:**
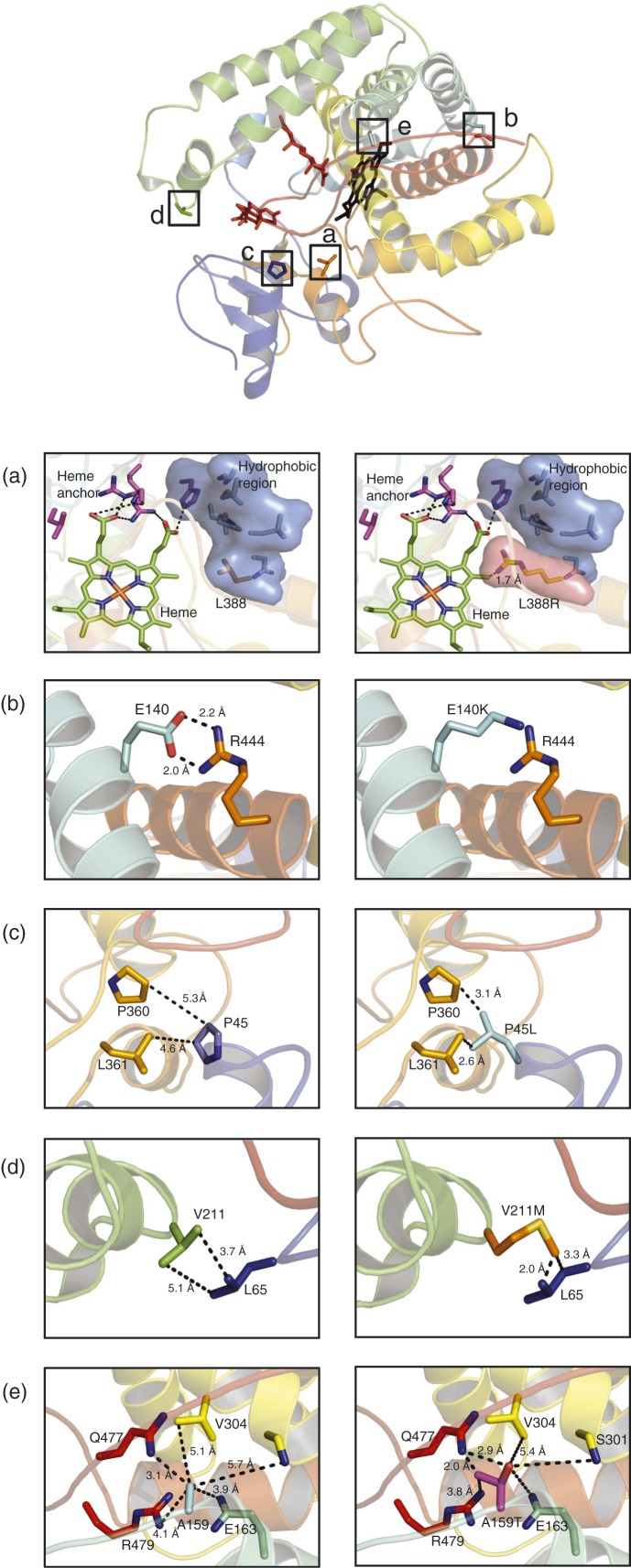
Tertiary structure of 21-hydoxylase with positions of five novel variants generated by PyMOL v.3.1. (a) p.L388R, (b) p.E140K, (c) p.P45L, (d) p.V211M and (e) p.A159T.

**Figure 2:**
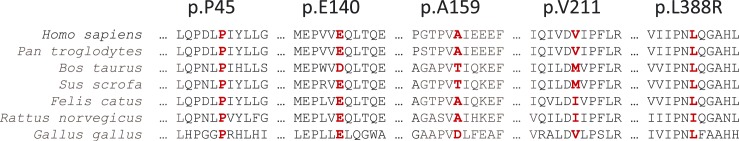
Amino acid sequence alignments of 21-hydroxylase for the p.P45, p.E140, p.A159, p.V211 and p.L388 residues for seven species.

The structures of 21OH and the novel variants based on the humanised model (15) are illustrated in Fig. 1. For p.L388R, the shift from the hydrophobic leucine to a negatively charged arginine in position 388 will probably lead to a disruption in a hydrophobic pocket surrounding the heme group (Fig. 1a). This is a highly conserved region (Fig. 2), and the p.L388R mutation was predicted as pathologic (Table 3).

The p.E140 residue forms a salt bridge with the R444 residue, and the shift from negatively charged glutamic acid to positively charged lysine breaks the salt bridge (Fig. 1b). A negatively charged amino acid in this residue is highly conserved (Fig. 2); however, PolyPhen2 predicted this mutation as possible damaging, while PROVEAN predicted it as neutral (Table 3) in line with significant residual enzyme activity (see above).

p.P45 is located early in the A helix (Fig. 1c), which is a highly conserved region (Fig. 2). The proline-to-leucine shift in position 45 was predicted as pathologic by both PolyPhen2 and PROVEAN (Table 3), but *in vitro* enzyme activity was normal.

The p.V211 residue (Fig. 1d) may interact with L65 through hydrophobic interactions and is located in a semiconservative region (Fig. 2). The shift from valine to methionine was predicted as benign/neutral (Table 3) in line with normal enzyme activity.

The variant p.A159T (Fig. 1e) is located on the surface of the 21OH protein. Sequence alignment shows that the alanine-to-threonine shift in position 159 is equal to the sequences of *B. taurus* and *S. scrofa* 21OH (Fig. 2), and was predicted as harmless (Table 3), again in line with a normal enzyme activity.

## Discussion

In the present study, we introduced a novel method for functional studies of *CYP21A2* mutants. Using the coupled transcription/translation system for protein expression, we were able to measure the 21OH enzyme activity without the need of cell culture facilities and radiolabelling substrates, which is the current standard method (19, 20, 21, 22). We then characterised five novel and three known variants of the *CYP21A2* gene found in patients with CAH (7) and AAD. Overall, the measured enzyme activities and structural simulation corresponded to the phenotype with some exceptions.

The three polymorphisms used for verification of the *in vitro* enzyme assay behaved as expected; p.R102K (23) showed no reduction in activity, whereas the well-known SV mutation p.I172N had <2% activity and the G291R showed no activity at all, in accordance with previously reports (24, 25, 26). The novel p.L388R mutation showed almost complete abolishment of enzyme activity *in vitro*, which is in total agreement with the SW phenotype and the structural simulation. p.L388 is a crucial residue as it interacts with the heme prosthetic group through hydrophobic interactions. When the leucine at this position is replaced by the positively charged and more space-demanding arginine, the heme group is restricted from its normal binding to 21OH, which would have deleterious consequences for the enzymatic activity as well as the three-dimensional folding of 21OH.

The patient with the p.E140K mutation was diagnosed as SW, but even if *in vitro* enzyme assay showed a significant reduction in 21OH activity (11%), it was still much higher than expected for the SW phenotype. In addition, the prediction tools did not foresee a severe damaging effect, and was more in line with a NC phenotype. However, structural analysis shows that the p.E140K disrupts a salt bridge with p.R444, which is crucial for maintaining the tertiary structure (15) that could explain the SW phenotype. Thus, prediction tools alone are not always précis (27). On the other hand, phenotype and genotype divergence in classical CAH has been described, and the same mutation can give different phenotypes even in siblings (28).

The variant p.P45L was detected in a SV patient, but the *in vitro* enzyme activity did not correlate to such a severe phenotype. However, this variant was predicted as probably damaging/deleterious by computer modelling. The p.P45L residue is located in the N-terminal region of the enzyme close to the hydrophobic domain that anchors 21OH to the endoplasmic reticulum (ER) membrane (15). The importance of the far N-terminal region has been experimentally determined earlier with regards to membrane targeting and insertion, as well as *in vivo* protein stability (29). It has also been demonstrated that the first 20 amino acids are necessary for membrane integration but that enzymatic activity *in vitro* can still be retained when these residues are deleted (30). Moreover, the p.P45 residue marks the start of an alpha helix that bends the direction of the polypeptide chain back towards the membrane. It is possible that this bending, for which proline in the 45 position is mandatory, is crucial for the correct orientation of 21OH in the ER membrane. If p.P45 is replaced by another amino acid, the overall stability and *in vivo* enzymatic activity of the protein may be severely decreased. Another possibility is that the removal of the proline residue at position 45 alters the enzymes orientation to the ER membrane and somehow disturbs the electron transfer mediated *in vivo* by the membrane-bound CYPOR. As we added exogenous CYPOR in our *in vitro* assay, this effect could be lost. However, it is still possible that the rest of the enzyme maintains its structural integrity and that the intrinsic enzymatic function is intact. Similar effects of mutated proline residues have been described for other proteins, such as cubilin (the intrinsic factor-B12 receptor) or the guanylate cyclase-activating protein 1 (involved in central vision) (31, 32). As we have carried out the functional characterisation of the p.P45L mutant in the absence of membranes, the severely reduced *in vivo* activity relative to wt 21OH could be missed in our system. We therefore propose that future studies should compare our novel method using cell-free expression of 21OH with the traditional use of transiently transfected COS7 cells, preferably with LC–MSMS-based quantification of steroids. In addition to the p.P45L mutant, such a comparative study should include a wide range of known and well-characterised 21OH mutations and variants.

The patient with the p.V211M variant was classified with a phenotype between SV and NC CAH. She also carried the NC CAH mutation p.V281L on the same allele, whereas the second allele was deleted. The *in vitro* 21OH activity of p.V211M was similar to the wt; thus the cause of the disorder might be from the p.V281L mutation, which actually has been reported with SV phenotype (6). An almost similar mutation, p.V211L, has been described previously in a patient with NC CAH together with p.V281L, and was suggested as normal based on no conservation between four mammalian species (33). Structural analysis shows that p.V211 is a part of the substrate cavity site and that it probably interacts with L65 through hydrophobic interactions (15). The conversion of valine into methionine at this position may alter this interaction slightly, but probably not to a damaging degree. PolyPhen2 and PROVEAN predicted p.V211M as benign/neutral, but with a score close to the threshold. It is unclear whether this mutation contributes to the intermediate form of SV/NC CAH due to a synergistic effect, similarly that has been seen in other studies were mild NC variants together have given SV (20, 21, 22, 34, 35) or SW phenotypes (36).

Finally, the p.A159T variant showed no reduction in the enzyme activity *in vitro*. This variant was found in heterozygosity in three patients with AAD and in one healthy subject, and was predicted as harmless. The p.A159 residue is located on the surface of the protein in a region outside the secondary structure motifs and away from the active site, and occurs naturally in other mammals such as pigs and cows (Fig. 2). The substitution of alanine with threonine is also common in homologous proteins from related species.

In conclusion, the *in vitro* enzyme assay and structural simulations revealed good correlations to the most severe phenotypes. For the other variants, a heterogeneous picture was observed. Nevertheless, *in vitro* enzyme assays and structural simulations are valuable supplements for evaluating the severity of novel *CYP21A2* variants.

## Figures and Tables

**Table 1:** Clinical description of the CAH patients with novel variants in *CYP21A2*.

**Variant**	**Clinical phenotype**	**Age of diagnoses**	**Treatment**
p.L388R	Salt wasting	Newborn	Mineralocorticoids
Clitoris hypertrophy	Glucocorticoids
p.E140K	Salt wasting	1 week old	Mineralocorticoids
Glucocorticoids
p.P45L	Simple virilising	4 years	Glucocorticoids
	Clitoris hypertrophy		
p.V211M+p.V281L	Simple virilising/non-classical CAH	21 years	Glucocorticoids
	Pubes growth and pronounced hirsutism from 7 years old		

**Table 2:** Primers used in mutagenesis.

**Mutations**	**Sequences** (5′→3′)
p.P45L	F: CAGCCCGACCTCCTGATCTATCTGCTTGG
	R: CCAAGCAGATAGATCAGGAGGTCGGGCTG
p.R102K	F: CAAGCTGGTGTCTAAGAACTACCCGGAC
	R: GTCCGGGTAGTTCTTAGACACCAGCTTG
p.E140K	F: CATGGAGCCAGTGGTGAAACAGCTGACCCAGGAG
	R: CTCCTGGGTCAGCTGTTTCACCACTGGCTCCATG
p.A159T	F: GGCACCCCTGTGACCATTGAGGAGG
	R: CCTCCTCAATGGTCACAGGGGTGCC
p.I172N	F: CACCTGCAGCATCAACTGTTACCTCACC
	R: GGTGAGGTAACAGTTGATGCTGCAGGTG
p.G291R	F: CTGCAGTGGACCTCCTGATCAGGGGCACTGAGACCACAGCAAAC
	R: GTTTGCTGTGGTCTCAGTGCCCCTGATCAGGAGGTCCACTGCAG
p.L388R	F: GTCATCATTCCGAACAGACAAGGCGCCCACCTG
	R: CAGGTGGGCGCCTTGTCTGTTCGGAATGATGAC

**Table 3:** Enzyme activity (% of WT) from four to five different experiments and *in silico* prediction of *CYP21A2* mutations and variants.

**Mutant/variant**	**% Average enzyme activity** (s.d.)	**Phenotype**	**Predicted phenotype** (score)	
PolyPhen2	PROVEAN^a^
p.K102R	119.7 (22.5)	Normal	Benign (0.001)	Neutral (0.722)
p.A159T	126.6 (29.9)	Unknown	Benign (0.023)	Neutral (0.36)
p.V211M	99.5 (32.4)	SV/NC^b^	Benign (0.029)	Neutral (÷1.019)
p.I172N	1.6 (0.8)	SV	Probably damaging (0.998)	Deleterious (÷5.499)
p.P45L	105.0 (10.6)	SV	Probably damaging (1.0)	Deleterious (÷5.067)
p.G291R	0^c^	SW	Probably damaging (1.0)	Deleterious (÷4.637)
p.E140K	11.3 (2.4)	SW	Possibly damaging (0.49)	Neutral (÷1.208)
p.L388R	1.1 (0.6)	SW	Probably damaging (0.986)	Deleterious (÷4.995)

NC, non-classical CAH; SV, simple virilising; SW, salt wasting.

a Threshold as deleterious set to <÷2.5.

b Phenotype of p.V211M+p.V281L/deletion genotype.

c Production of 11DOC concentration under the detection limit of LC–MSMS method.
